# Barriers and facilitators to narrative nursing implementation for junior nurses: A qualitative study

**DOI:** 10.1097/MD.0000000000039922

**Published:** 2024-10-04

**Authors:** Li Zhang, Qiang Han, Lin Nan, Huiyun Yang

**Affiliations:** a Department of Geriatric Digestive Surgery, The Second Affiliated Hospital of Xi’an Jiaotong University, Xi’an, Shaanxi Province, China; b Cancer Center, The First Affiliated Hospital of Xi’an Jiaotong University, Xi’an, Shaanxi Province, China; c Nursing Department, The Second Affiliated Hospital of Xi’an Jiaotong University, Xi’an, Shanxi Province, China.

**Keywords:** barriers, facilitators, junior nurses, narrative nursing, qualitative study

## Abstract

This study aimed to explore the experiences of barriers and facilitators to the implementation of narrative nursing by junior nurses. Participants were recruited using purposive sampling. Semi-structured interviews were conducted from October 2023 to February 2024. Data were thematically analyzed using the Colaizzi seven-step method. Five barriers and 3 facilitators to the implementation of narrative nursing by junior nurses emerged in the study. Relevant barriers include lack of practical skills related to narrative nursing, neglect of the concept of humanistic care, negative events, high workload for junior nurses, restricted implementation environment. The facilitators contain patients’ and family members’ trust, training related to knowledge and skills, as well as harmonious and cooperative working atmosphere. There is still room for improvement in the implementation of narrative nursing for junior nurses, and the systematic training of narrative nursing knowledge and skills should be enhanced; the medical humanistic environment should be optimized; and the psychological resilience of junior nurses should be improved, so as to encourage them to actively implement narrative nursing at the early stage of their careers, and to deepen the high-quality development of nursing humanism.

## 1. Introduction

Narrative nursing is a method of nursing practice based on the theory of narrative medicine, in which nurses externalize and reconstruct problems by listening to and absorbing patient’s narratives, helping them to achieve positive self-transformation.^[1,2]^ The implementation of narrative nursing can effectively alleviate patients’ psychological pain, improve patients’ quality of life, and further improve nurse–patient relationship. In addition, it can also help nurses to improve the level of humanistic literacy and reduce compassion fatigue and burnout.^[2,3]^ Narrative nursing, as a new way of humanistic nursing, effectively compensates for the lack of humanistic care.

The junior nurses are in the early stage of their career and have high professional enthusiasm, but due to insufficient experience, they are more prone to focusing on the completion of the relevant treatments and neglect the observation of the changes in the patient’s mood and the implementation of narrative nursing.^[4]^ Study has shown that strengthening the implementation of narrative nursing care for junior nurses can not only improve the quality of clinical nursing services but also help them discover their professional value and reduce attrition rate.^[5]^ However, few studies have been conducted to explore barriers and facilitators to narrative nursing implementation for junior nurse from a qualitative perspective.

Constructivist theory, which emphasizes that knowledge is constructed through an individual’s interaction with the environment, can be used to explore how junior nurses understand and respond to narrative nursing implementation facilitators and barriers based on their own experiences, attitudes, and environmental factors.^[6]^ Therefore, this study aimed to capture relative facilitators and barriers regarding narrative nursing implementation among junior nurses via semi-structured interviews under the guidance of constructivist theory and to provide evidence and reference for promoting narrative nursing practice among junior nurses.

## 2. Methods

### 2.1. Study design

This study was conducted from October 2023 to February 2024 at a tertiary grade A hospital in Xi’an, Shaanxi Province, China. Junior nurses of this hospital were selected for the study based on maximum variation strategy.^[7]^ Inclusion criteria: with bachelor’s degree; clinical experience ≤5 years^[8]^; implemented narrative nursing for patients; and consent to participate in the study. The sample size was determined on the basis that the information from the participants’ interview data was repeated and no new themes emerged. A total of 16 nurses were included in this study.

### 2.2. Data collection

A descriptive phenomenological research method was used to conduct semi-structured interviews with junior nurses. According to the interview outline, the junior nurses were made to narrate within a specific scope. The interviewers, comprising a charge nurse and a director of nursing, both of whom are highly qualified female professionals with a master’s degree and professorship respectively, introduced the purpose, method, content, and confidentiality principles of the interview in detail to the participants, ensuring their understanding and cooperation. Face-to-face interviews were conducted after consent was obtained and recorded in the form of transcripts. The interview time for each junior nurse was 30 to 60 minutes, guiding the junior nurses to speak more comprehensively about the real views and experience of the facilitators and barriers to the implementation of narrative nursing, with the researchers focusing on listening and understanding, avoiding inducing hints to the interviewees, and appropriately reviewing, confirming and summarizing the interview content, clarifying the ambiguous information, and improving the accuracy of the data.

### 2.3. Data analysis

Nvivo 12.0 software was used to conduct data analysis. When the interviews were completed, 2 researchers completed the transcription of the audio recordings within 24 hours and supplemented them with the contents of the transcripts. The process followed a 7-step procedure developed by Colaizzi,^[9]^ whereby themes were analyzed, collated, summarized, and distilled, and where disagreements arose, group discussions were held until a consensus was reached, with interviews and data analysis taking place in parallel.

### 2.4. Ethical considerations

Ethical clearance was obtained from the ethics committee of the hospital where the study was conducted. The purpose of the study was explained to the participants in detail, and they were free to withdraw from the study at any time. An informed consent form was signed by all participants before starting the interview. Anonymity and confidentiality anonymity and confidentiality were also assured. All data and transcripts were de-identified.

### 2.5. Qualitative rigor

All members of the research group have received training in qualitative research, mastering qualitative research methods and interview techniques. During the data collection process, the researchers transcribed the data in a timely manner, repeatedly confirmed any uncertain information with the interviewees, and made corrections to the transcribed text to ensure the accuracy and reliability of the data. Non-verbal messages from each participant were observed and recorded on-site. Transferability was ensured through vivid descriptions of findings and links to previous research. Data analysis was completed by 2 researchers, and the research team discussed together to determine the final coding and themes.

## 3. Results

In the study, all the junior nurses had a bachelor’s degree. The mean age was 25.50 ± 2.10. And 2 of them were male and 14 (87.5%) were female. The mean number of years of working experience was 3.19 ± 1.32.

Data saturation was reached after 16 interviews were conducted. Each interview lasted 30 to 60 minutes, with an average of approximately 50 minutes. Two themes were extracted as barriers and facilitators.

### 3.1. Barriers to the implementation of narrative nursing

#### 3.1.1. Lack of practical skills related to narrative nursing

Some interviewees reported that they could perceive the advantages of narrative nursing, but their own narrative nursing skills were insufficient. Although they had learned the relevant theoretical knowledge at school, they did not really understand it and could not really implement narrative care in practice.

*I learned narrative nursing at school, but I simply thought it was storytelling and didn’t understand it well. There are so many patients in the clinic who need psychological counselling, but I lack the knowledge and skills in this area*. [N11]

*Once, the patient I was in charge of was having surgery the next day, but he seemed particularly anxious, repeatedly asking how long it would take to recover, how much it would cost, and whether he would get better after the surgery, etc. But I didn’t know how to comfort him. But I didn’t know how to comfort him. I felt like I had learned about this, but my mind went blank when it came time to use it. (Sighs helplessly*). [N12]

The junior nurses also indicated that they have mastered basic clinical communication skills, but may be confused or unable to cope with complex situations due to inadequate communication skills, such as lack of confidence, easy to be nervous and anxious, or even at a loss for words when dealing with doctor-patient disputes, or communicating with the family members of deceased patients.

*When some patients or family members are in a particularly bad mood, I am afraid to communicate with them. I worry that if I say something wrong to them, they will complain about me or distrust me even more*. [N10]

*I have no problem with basic communication with patients. But when it comes to doctor-patient disputes, or when the family members of a deceased patient need to be communicated with, I am more anxious and especially nervous, and I don’t even know how to communicate appropriately*. [N16]

#### 3.1.2. Neglect of the concept of humanistic care

The junior nurses generally have a positive will to implement narrative nursing, but in the actual clinical work, due to the general concept of “emphasizing technology and neglecting humanism,” the patients and their families pay more attention to the technical ability of the junior nurses, resulting in the junior nurses neglecting the concept of humanistic care in the clinical work.

*Most patients place more emphasis on therapeutic nursing tasks, resulting in the first thing to be done in the clinic being intravenous fluids, nebulized inhalation, and other such operations. So it seems to me that humanistic or narrative nursing has become an optional rather than a mandatory task*. [N1]

*When I first started to work, I was also enthusiastic. I would help patients whenever they needed help, and I was willing to communicate more with patients to understand their stories of illness. But then I realized that even though I cared about the patients, they recognized the teachers with good technical operation more*. [N5]

#### 3.1.3. Negative events reduce willingness to implement narrative nursing

From a perspective of career development, experiences in healthcare environments can trigger reflection on future careers for junior nurses. In particular, the negative events encountered put mental stress on the junior nurses, leading to avoidance of humanistic care, which in turn affects the belief in the implementation of narrative nursing.

*A patient with a medical dispute was hospitalized on the unit and I had witnessed his family members assaulting the attending doctor. I was afraid to go to his hospital room during that time, to communicate with him, much less implement narrative care*. [N5]

*Any time a medical professional makes a bit of a mistake, they are exposed in the media and even on the internet*. [N9]

#### 3.1.4. High workload for junior nurses

Most of the junior nurses expressed that the high workload of clinical care resulted in their communication to patients being more on the technical level and less on in-depth emotional communication.

*Whenever I go to work, it feels like there is an endless amount of work to do. Even if I am not very busy, I feel tired and want to take a break. I don’t reach out to patients unless they are going to talk to me*. [N6]

*When I work the night shift alone, I am as busy as a bee. If a patient needs to tell me his story, I certainly don’t have the time, much less the time to listen carefully, and if I do, I assume the other patients will complain*. [N11]

#### 3.1.5. Restricted implementation environment

Interviewees mentioned that limited settings were also an important barrier to implementing narrative nursing.

*Sometimes I want to relieve the patient’s anxiety through narrative nursing, but there are constantly passing doctors and nurses, as well as other patients and family members, and I am often interrupted in the middle of the process, and the effect will be greatly reduced if I continue afterwards. This environment is not conducive to in-depth communication, and it is not easy for the patient to relax and open up to you to say something from the heart*. [N12]

*Although there are empty, quiet rooms, some patients need to be in their own rooms for oxygen and other treatments, and it’s not appropriate for you to ask them to come to an empty room. And in case he has an emergency I may not be able to save him in time, and if something does happen the responsibility will probably lie with me*. [N14]

### 3.2. Facilitators of the implementation of narrative nursing

#### 3.2.1. Patients’ and family members’ trust in junior nurses

Some junior nurses indicated that they would like to gain the support and trust of patients and their families when they implement narrative nursing. Because their self-confidence is undermined in a work environment that lacks basic trust, and they are subsequently reluctant to implement narrative nursing.

*I would like to receive more trust and support from my patients and their family members, which would encourage me to implement narrative nursing*. [N7]

*Some patients and family members treated me differently than senior nurses, I could feel that they thought I was less experienced and didn’t trust me. I’ve even heard family members chat that I’m a young nurse and don’t have the knowledge they want to ask for, so I don’t have much of a chance to interact with my patients, let alone implement narrative nursing*. [N2]

#### 3.2.2. Training related to knowledge and skills of narrative nursing

The junior nurses had very little training in narrative nursing after being employed. Some of the junior nurses also indicated that their senior clinical instructors also lacked in-depth understanding of the knowledge and skills of narrative nursing. They hoped that more relevant training would be conducted in the future.

*After admission to the hospital, the continuing education curriculum favoured basic nursing operations and knowledge of specialty care, with a small portion of humanistic nursing training involved and almost no narrative nursing training*. [N10]

*Sometimes tumour patients lose confidence in life. I have seen senior nurses give examples to a patient, saying that they have seen patients who are especially cooperative with the treatment and recovered well, and the patient feels hopeful and confident after listening to them. Maybe the nurses didn’t know that this is narrative nursing, in fact, a lot of them did this in the clinic. But more standardized training is needed in the future*. [A7]

#### 3.2.3. Harmonious and cooperative working atmosphere

The working atmosphere of the hospital and the department affects the cohesion and collaboration of the entire team. The implementation of narrative nursing will be smoother and more effective when all members of the department are committed to providing humanistic care for patients. Harmony in hospitals and departments is not only between nurses and patients, but also between doctors and nurses. Tension can lead to a poor humanistic atmosphere, and the motivation of nurses to implement narrative care for patients will be greatly reduced.

*Recently, I have done narrative nursing for patients, and I feel that it is quite useful to the patients, and I also feel very happy. Unlike my previous department, there were often conflicts between colleagues, and when something happened, they would blame each other and no one would do narrative nursing for the patients*. [N2]

*Sometimes I can’t think of doing narrative care because my coworkers around me don’t do it either*. [N7]

## 4. Discussion

The objective of this study was to analyze the barriers and facilitators to narrative nursing implementation for junior nurses. In China, the concept of narrative nursing has not yet been popularized, and even though a small number of medical schools have offered a compulsory course on narrative nursing, it is mainly open to postgraduate students, and there is still a status quo of fewer hours and an unscientific curriculum system.^[10]^ While junior nurses achieved their pedagogical goals during the theoretical education phase of narrative nursing in school, they neglected the comprehensive formation of their knowledge structure and competencies, and how to extend it to clinical practice. A study by Zhang^[11]^ showed that the integration of close reading training into professional nursing courses is a feasible teaching method to improve nursing professionals’ narrative skills, empathy, and humanistic caring skills.

In addition, the interviews revealed that the most significant barrier to narrative nursing implementation among the junior nurses was the lack of narrative nursing capacity, and the junior nurses who had been introduced to narrative nursing had a more positive attitude toward its implementation, which are similar to the findings of Su and Wang.^[12]^ This may be due to the fact that narrative nursing education in China has not yet established a mature system and lacks a targeted and systematic training model in practice. However, junior nurses who have encountered narrative nursing feel its value and advantages in clinical practice and show a positive willingness to practice it.^[10]^ Nursing administrators and educators should first clarify that the core of narrative nursing education lies in practice. During narrative nursing education and training, an integrated training system can be established at both the hospital and school levels, and narrative scenes can be jointly formulated by integrating all the educational resources; schools can organize nursing students to carry out role-playing activities for the characters in the narrative content to enhance the combination of theory and practice.^[11]^ In addition, rest time can be used to carry out hospital volunteer service work. Through close contact with patients and listening to their stories of illness, nursing students are prompted to have completed the reflective practice process of narrative nursing when entering clinical practice, laying a solid foundation for the implementation of narrative nursing.^[13]^ Nursing administrators in hospitals and nursing educators at schools should maintain close cooperation, make full use of clinical resources to deepen the expansion of the content of narrative nursing education; Nursing administrators in hospitals and nursing educators at schools should maintain close cooperation, make full use of clinical resources to deepen the expansion of the content of narrative nursing education; conduct regular feedback surveys on nurses’ narrative caring competence, and form a closed-loop training model of narrative nursing knowledge-attitudes-practices, so as to create the necessary conditions for its implementation.

The results of the interviews showed that junior nurses’ willingness to narrative nursing decreased after experiencing negative events, which is similar to the results of Fu and Xiong’s study.^[14]^ The reason for this may be related to the low psychological resilience of junior nurses, who have just stepped into society from school and cannot quickly adapt to the complex environment of the hospital. Their psychological adjustment mechanism after facing negative events has not yet been fully established, coupled with the concept of “emphasizing technology and neglecting humanism,” which leads to challenges in the implementation of narrative nursing.^[15]^ Psychological resilience refers to an individual’s ability to adapt well in the face of stress, adversity, trauma, or threat, and to recover quickly and cope successfully with difficulties when exposed to major stresses and dangers with a positive attitude.^[16]^ Evidence suggests that nurses who have good psychological resilience are more capable of coping with negative events and reduce the psychological trauma caused by these events.^[17]^ Increased psychological resilience not only enhances nurses’ confidence in narrative nursing, but also contributes to increased acceptance and willingness to practice narrative nursing.^[17]^ Therefore, it is recommended that in the training and career development of junior nurses, nursing administrators should strengthen the cultivation and support of psychological resilience, advocate the concept of humanistic care, and firmly establish the belief in narrative nursing, so as to lay the foundation for the implementation of narrative nursing.

Nurses’ occupational environment contains various elements such as physical and social dimensions, which directly or indirectly affect the professional behavior of nurses.^[18]^ Improving nurses’ occupational environment can significantly improve nurses’ humanistic competence and facilitate the implementation of narrative nursing. The results of the interviews revealed that junior nurses face a lack of external environmental support in narrative nursing practice, which is similar to the results of the study by Liu et al.^[19]^ The implementation of narrative nursing in a warm, comfortable, and safe place with privacy is more convenient for in-depth communication and interaction between nurses and patients. However, in actual clinical work, its implementation can also not be limited to any place, and nurses should fully seize the opportunity to communicate with patients to achieve the purpose and effect of narrative care.

Moreover, participants pointed out that patients’ and their family members’ trust in the junior nurses and a harmonious and cooperative work atmosphere would promote the implementation of narrative nursing. It is suggested that nursing administrators help junior nurses build a humanistic professional environment,^[20]^ create a workforce atmosphere full of humanistic care, and improve job satisfaction and peer support among junior nurses.^[21]^ It is also useful to utilize the power of the media to enhance social respect and support for junior nurses’ work. With the rapid development of information technology, it is extremely important to improve nurses’ ability to acquire information through online learning platforms.^[22]^ Nursing administrators should make full use of network resources to accelerate the popularization of narrative nursing by regularly posting real cases of narrative nursing on WeChat official accounts (a platform for Chinese users to access information and services), creating a positive learning environment, and providing support and guarantee for the development of narrative nursing. Additionally, multidisciplinary collaboration for group narrative training can increase the intersection of different disciplines, thus promoting a diversity of narrative contexts, which in turn makes narrative nursing training for junior nurses more authentic and profound.^[23]^

## 5. Limitations

In this study, one potential limitation is the relatively small and homogeneous sample size, consisting only of junior nurses from a single tertiary hospital in China. This limits the generalizability of the findings to other healthcare settings or regions. Additionally, the study relies on self-reported data from semi-structured interviews, which may introduce bias as participants might have provided socially desirable responses. Furthermore, the study does not explore the perspectives of other key stakeholders, such as senior nurses, patients, or administrators, which could provide a more comprehensive understanding of the factors influencing the implementation of narrative nursing. Future research should consider incorporating a larger and more diverse sample and include multiple stakeholders to validate and expand on these findings.

## 6. Conclusion

In this study, 16 junior nurses were interviewed in depth to explore their experiences of barriers and facilitators to the implementation of narrative nursing. Relevant barriers include lack of practical skills related to narrative nursing, neglect of the concept of humanistic care, negative events, high workload for junior nurses, restricted implementation environment. The facilitators contain patients’ and family members’ trust, training related to knowledge and skills, as well as harmonious and cooperative working atmosphere. It is recommended that nursing administrators strengthen the educational cooperation between medical schools and hospitals, improve the psychological resilience of junior nurses, build a humanistic occupational environment, promote the implementation of narrative nursing by junior nurses, and help promote the widespread application of narrative nursing in clinical settings.

## Acknowledgments

We would like to thank the participants for their time and efforts in this study. We are very grateful for the support and help from The Second Affiliated Hospital of Xi’an Jiaotong University.

## Author contributions

**Conceptualization:** Li Zhang, Qiang Han, Lin Nan, Huiyun Yang.

**Data curation:** Li Zhang, Qiang Han.

**Formal analysis:** Li Zhang, Lin Nan.

**Methodology:** Li Zhang, Qiang Han, Lin Nan.

**Software:** Li Zhang, Qiang Han.

**Visualization:** Li Zhang.

**Writing – original draft:** Li Zhang, Qiang Han, Lin Nan.

**Writing – review & editing:** Li Zhang, Qiang Han, Lin Nan, Huiyun Yang.

**Supervision:** Huiyun Yang.
